# The Promotion and Development of Community Health for Personal Health: Theories and Applications

**DOI:** 10.3390/healthcare13070747

**Published:** 2025-03-27

**Authors:** Yang Gao, Ya Sun, Yu-Hsi Yuan, Chia-Huei Wu, Datian Bi

**Affiliations:** 1 International Business School, Hainan University, Haikou 570228, China; 996139@hainanu.edu.cn (Y.G.); sunya_stu07@163.com (Y.S.); 2 Department of Graphic Communication, Taiwan University of Arts, Taipei 11114, Taiwan; 3 Department of Hotel Management and Culinary Creativity, Minghsin University of Science and Technology, Hsinchu 30401, Taiwan; 4 School of Management, Jilin University, Changchun 130022, China; bdt@jlu.edu.cn

## 1. Introduction

The post-COVID global health landscape has undergone profound transformation, with community health development emerging as a cornerstone of sustainable progress. The WHO Healthy Cities Program now strategically positions communities as critical domains for achieving health equity, leveraging participatory governance models and cross-sectoral resource allocation to establish inclusive health ecosystems [1]. Health can be understood as a state of dynamic equilibrium between the mental and physical condition, and by integrating the two, holistic approaches to disease prevention and health promotion can be achieved [2,3]. Community wellness initiatives function as catalytic platforms, empowering health professionals to engage residents in participatory research, policy co-creation, and targeted health promotion campaigns. These programs demonstrate particular efficacy in mitigating health disparities among marginalized populations by addressing structural barriers rooted in social determinants of health [4,5]. Empirical evidence confirms their dual capacity as advocacy instruments and intervention vehicles. Population-based studies consistently validate the vital role of community health initiatives in enhancing collective well-being, particularly through community-driven solutions that effectively reconcile cultural contexts with evidence-based practices.

Against this background, this topic, “**The Promotion and Development of Community Health for Personal Health: Theories and Applications**”, focuses in depth on the integration of resident autonomy, community intervention, collaborative governance, and sustainable development in community health building. Through multidimensional perspectives, it explores how community health governance models can establish inclusive, resilient, and sustainable health ecosystems. The included studies employ theoretical frameworks, empirical analyses, and policy evaluations to demonstrate the key factors that critically affect a model’s success in enhancing population health outcomes—autonomy, systematization, and adaptation. This collection advances academic discourse by proposing novel analytical paradigms while offering concrete guidance for evidence-based policy formulation and professional practice. The dual emphasis on theoretical innovation and practical implementation charts new pathways for transforming community health governance in the post-pandemic era, bridging the gap between conceptual understanding and practical application in community health systems.

## 2. Participatory Design: Bridging Access and Autonomy

Participatory design is reshaping the boundaries of population health autonomy, inspiring self-determination, autonomy, and self-help for self-health management [6]. This topic reveals the critical role of participatory design in reconfiguring population health autonomy through empirical research across multiple geographic regions and scenarios. The series of studies included focuses on a variety of topics, such as disease management, nutritional interventions, and drug information sharing, and provides important policy implications for residents’ self-health management.

In the field of chronic disease management, Asma et al.’s paper, “*The Development of a New Tool to Help Patients and Their Providers Evaluate Self-Management of Type 2 Diabetes Mellitus*”, empirically analyzes the correlation between knowledge level, education level, and self-management behaviors of diabetic patients by conducting a cross-sectional study of 306 adult type 2 diabetes mellitus (T2DM) patients (Contribution 1). Significant positive correlations were found between knowledge of diabetes and self-management efficacy, particularly among higher-educated subgroups. The novel assessment tool developed in this study can be used to provide personalized management advice to patients and healthcare providers, helping to optimize clinical decision-making. Guyue’s paper, “*Japanese Consumers’ Attitudes towards Obtaining and Sharing Health Information Regarding Over-the-Counter Medication: Designing an Over-the-Counter Electronic Health Record*”, examines consumer perspectives on OTC-EHR adoption, health app usage, and anonymous data sharing via an online survey (Contribution 2). The study identified three key determinants of OTC-HER adoption: e-health literacy, perceived utility, and privacy concerns. These findings can be used to inform OTC-EHR design in order to enhance self-medication safety and risk mitigation. Furthermore, psychological barriers to the sharing of anonymized health data can be addressed through expanded OTC-EHR implementation and optimized information interfaces. Strategic system designs empower consumers to transition from passive medication users to proactive health managers.

In addition, digital interventions demonstrate significant potential in shaping student dietary behaviors [7,8]. Laura et al.’s paper, “*Social Media for Nutrition Education-A Randomized Controlled Trial to Promote Fruit and Vegetable Intake in a University Setting: The University of Valladolid Community Eats Healthy Study*”, describes a randomized controlled trial, involving 211 Spanish university adults, conducted to assess the efficacy of the UVEH program (Contribution 3). The intervention group demonstrated higher vegetable consumption and greater fruit intake compared to controls utilizing standard virtual campus tools. The structured and interactive content format of the tested digital health platform, extending far beyond the capabilities of social media platforms, was effective in improving health behaviors. These findings validate digital health platforms as effective tools for encouraging behavioral changes, offering a framework for implementing “tech-education” models in community health initiatives. The results underscore the necessity of transitioning from passive nutritional guidance to active digital engagement strategies for enhanced community health management. The study “*Effect of a Multicomponent Food Pantry Intervention in Client Subgroups*”, by Jenny et al., analyzed 193 Minnesota adults through a cluster-randomized trial to examine whether the Superfood Shelf program differentially impacted diet quality across demographic subgroups (Contribution 4). The results demonstrated that the program had consistent effectiveness in improving nutritional intake, regardless of gender, race/ethnicity, education, or employment status, confirming the intervention’s equitable benefits. This evidence supports the implementation of nutrition-focused SuperShelf programs to enhance accessibility to healthy food, while mitigating dietary disparities, in vulnerable populations.

Collectively, these studies illustrate how implementing a participatory co-design in community health systems can transform personal health autonomy. By incorporating digital health ecosystems with intelligent tools, such approaches can empower individuals to transition from passive healthcare recipients to engaged managers of their personal well-being.

## 3. Structural Interventions: The Role of Communities

Policy-driven structural reforms are essential for establishing sustainable community health ecosystems [9,10]. This topic draws on multi-country empirical studies to show that systemic interventions, implemented through institutional restructuring and optimization of resource allocation, can enhance personal health outcomes, while generating self-reinforcing developmental cycles at community levels.

In the domain of adolescent health management, José et al.’s study, “*Affective*–*Sexual Behaviors in Youth: Analysis of a Public Health Survey in the School Setting*”, employed a cross-sectional design and investigated fourth-grade Spanish adolescents to evaluate the need for structural educational reforms (Contribution 5). The analysis revealed significant gender disparities in affective–sexual literacy, with female participants demonstrating higher health knowledge retention and a greater capacity for persuasion regarding emotional and sexual behavior. These findings underscore the necessity of implementing comprehensive sexuality education programs, youth empowerment frameworks, and media literacy initiatives to combat misinformation. Such structural interventions advance urban public health outcomes through safer and healthier behavioral practices. The paper “*Impact of the COVID-19 Pandemic on the Use of Antidepressants by Young Adults in the ASL TO4 Regione Piemonte (Italy)*”, written by Lucrezia et al., describes an interrupted time series analysis of 1071 young adults in Piedmont, Italy, revealing the prolonged psychiatric impact of COVID-19 (Contribution 6). The analysis identified a sustained elevation in morbidity post week 134, reflecting cascading care delays and unmet mental health needs. This psychiatric morbidity trajectory suggests impending strain on pharmaceutical supply chains and community health resources, with projected 18–24-month lag effects in service demand. Public health events expose the vulnerability of community mental health services; thus, there is a need to strengthen community mental health screening and early intervention, and to build resilient support networks. Such measures align with the WHO’s Framework for Mental Health System Recovery (2023), offering scalable solutions to transform episodic crisis response into sustained mental health ecosystem resilience.

Turning our focus to the topic of care for the elderly, it is essential to recognize the growing need for comprehensive support systems that address the physical, emotional, and social well-being of the aging population. Marcia et al.’s paper, “*An Over-the-Counter Hearing Aid Clinical Trial in Rural Alabama: Project Design and Potential Implications for Pharmacy and Audiology Interprofessional Collaborations*”, which presents a clinical trial conducted in rural Alabama (n = 215), demonstrates the potential for structural innovation in primary care (Contribution 7). Intervention with over-the-counter hearing aids (OTC HAs) reduced the time taken to acquire hearing aids. However, some older participants required technical assistance with device activation, revealing critical age-related implementation barriers. These findings advocate for the establishment of an integrated pharmacy–audiology network to expand access to hearing healthcare in rural areas. Pharmacies can serve as access points for hearing healthcare in rural areas, offering assistance with device procurement and basic usage guidance. Strategic collaboration with licensed audiologists would enable tiered service delivery, optimizing resource allocation, while maintaining clinical oversight.

With regard to health management of the entire family, Shahidullah’s study, “*Women Agro-Entrepreneurship Promoting Vegetables at a Family Level: A Healthcare Approach towards Non-Communicable Disease Risk Reduction*”, employed a participatory action research design, investigating rural Bangladeshi households to identify the role of women in household dietary decision-making and its impact on the improvement of non-communicable diseases (NCDs) (Contribution 8). The study found that women play a central role in health management, such as in household dietary choices and disease prevention. However, they have limited decision-making power when it comes to vegetable production and consumption, and they have limited knowledge and awareness of NCDs. The findings suggest that empowerment interventions for women in terms of knowledge, skills, access to finance, and household decision-making power are beneficial in achieving household nutrition improvement and disease prevention. Therefore, community health strategies and policies to address non-communicable diseases should incorporate a family-centered approach and promote women’s agricultural entrepreneurship, which, in turn, promotes family-based health management.

Systematic career support is, likewise, a key part of structural change. Héctor et al.’s study, “*Cross-Cultural Adaptation and Validation of the Perceptions of Empowerment in Midwifery Scale in the Spanish Context (PEMS-e)*”, conducted cross-cultural adaptation of the empowerment assessment tool across 18 Spanish regions (Contribution 9). Analysis of 410 midwives revealed alarming workforce sustainability challenges: 25% of the midwives reported anticipating career attrition within six months, revealing critical empowerment deficits. The findings confirm that the PEMS-e is a valid tool for assessing the level of empowerment of midwives in Spain, and it can be used in future research and for the development of strategies to improve professional satisfaction and promote retention of midwives.

Collectively, cross-regional studies chart a course for structural transformation in community health. First, an institutional framework can be built using legislative and policy instruments. Second, spatial reconfiguration strategies can be used to integrate health resources into the community system. Finally, an occupational support system can be established to ensure service sustainability. The abovementioned studies offer methodological support for flexible resource deployment and for creating a form of ecosystem development that is conducive to community health.

## 4. Collaborative Governance: From Policy to Practice

Multisectoral partnerships serve as critical conduits for translating health policies into measurable outcomes. This issue demonstrates that policy efficacy hinges not merely on institutional architecture, but also, crucially, on the participation and collaboration of stakeholders. In the context of personal health, preventive care and chronic disease management should be emphasized, and strong vertical relationships with community physicians and health workers should be established, in order to achieve close collaboration across health and social care sectors [11,12]. Such collaborative mechanisms, through information sharing, reorganization of authority and responsibility, and technological innovations, can provide an efficient pathway from policy texts to actual health outputs [13]. Many authors have examined the involvement of subject entities from different perspectives.

In the realm of patient management and disease prevention, Lidi et al.’s paper, “*Shared Decision*-*Making to Improve Health—Related Outcomes for Adults with Stroke Disease*”, constructs a shared decision-making (SDM) framework for stroke rehabilitation (Contribution 10). Through the European ALAMEDA project, an SDM framework for stroke rehabilitation was developed, and an implementation pathway for collaborative governance was verified. Eleven representatives from diverse fields, such as doctors, nurses, patients, and caregivers, were invited to engage in a consultative process. Key patient data were collected and analyzed through a questionnaire survey, in order to formulate a set of general and specific guidelines for patients’ use of wearable sensing devices. Simultaneously, patient-specific needs and preferences were integrated into ALAMEDA’s design and development. This shows the significance of interdisciplinary cooperation in stroke patient rehabilitation. The paper “*Adherence to Pulmonary Tuberculosis Medication and Associated Factors Among Adults: a Cross-Sectional Study in the Metinaro and Becora Sub-Districts, Dili, Timor-Leste*”, written by Amentinho et al., analyzes medication adherence and key predictors among adults, based on a sample of 398 tuberculosis patients (Contribution 11). The study revealed that better healthcare service quality increased patients’ likelihood of high medication adherence. Regarding personal behavior, patients who drank alcohol or engaged in only occasional physical activity were much less likely to adhere well. Healthcare service quality and personal factors, like lifestyle behaviors and social stigma, were found to be crucial predictors of adherence to TB medication. Consequently, strengthening healthcare infrastructure, implementing multisectoral and collaborative behavior, and reducing social stigma are of particular importance. Additionally, mHealth technologies, such as text message reminders and telemedicine, could help to improve patients’ real-time medication adherence. Ines et al.’s study, “*What Do Younger and Well-Educated Adults Think about Self-Medication? Results of a Survey during a Public Science Event at Leipzig University*”, sampled 189 individuals from Leipzig University (Contribution 12). It aimed to explore participants’ attitudes and behaviors regarding personal self-medication (using over-the-counter medications). The study showed that young, well-educated people often practiced self-medication, and rated their own expertise highly. These findings support the idea of autonomous health governance in specific populations like this one. However, the study also emphasized that pharmacists and doctors remained the main information sources, even for self-medication. Thus, it is crucial to stress the importance of seeking guidance from healthcare professionals. The public, especially those who consider themselves “highly knowledgeable”, should be educated about potential risks. Moreover, public education on drug dependence and the adverse effects of medications needs to be strengthened. Jiaji’s paper, “*Campaign Governance and Playfulness: Unraveling Chinese HPV Immunization Promotion Efforts on Douyin*”, analyzes data from 73 short videos released by official media through a thematic approach and semi-structured interviews with 37 Chinese stakeholders (Contribution 13). The results indicate that gamified promotional strategies enhanced the popularity of the HPV vaccine campaign. However, a lack of multi-stakeholder partnerships and a single messaging format lessened the campaign’s effectiveness. These findings underline the necessity of strengthening multi-stakeholder partnerships to optimize the delivery of public HPV vaccination services. This study is not only useful in helping the global audience better understand China’s immunization campaigns, but also offers insights that may influence future global health promotion endeavors.

Moreover, with respect to dietary management, Karolina et al.’s paper, “*Nutrition in Educational Institutions—The Perspective of School Principals and Parents on the Tasks of Local Governments (Poland)*”, uses data from a sample of 200 school principals and 1000 parents in Poland (Contribution 14). It explores their perceptions of local governments’ roles in school nutrition management. The study results show significant differences between principals’ and parents’ satisfaction regarding meal quality, co-organization, and allergen information. This reveals the negative impact of information asymmetry on collaboration. Thus, there is a strong need to enhance communication and cooperation among schools, parents, and local governments, in order to boost the effectiveness of nutrition programs. This study suggests that regular information exchange activities could be organized to improve information flow, thereby increasing parent satisfaction and the overall effectiveness of school nutrition programs.

The results of the abovementioned studies indicate that community health governance does not rely on personal autonomy alone. Instead, it demands the involvement of multiple stakeholders to foster a sustainable community health model.

## 5. Focusing on Special Groups: Sustainable Health Governance

Sustainability and equity are fundamental to the effective implementation and long-term success of health interventions. For these interventions to have a lasting effect, they need to be thoroughly embedded in the local socio-cultural environment. When health initiatives are well integrated with the community’s internal resources, the “participation problem” faced by marginalized and disadvantaged groups can be effectively addressed, and thus, a robust and sustainable health support network can be established [14,15]. A set of studies centered on special populations, like the elderly, stroke patients, disabled individuals, and marginalized ethnic groups, offer empirical evidence supporting the sustainable development of community health governance. This is demonstrated across three aspects: service perceptions, resource accessibility, and cognitive restructuring.

For the elderly, optimizing their service perceptions is crucial. This requires beginning with accurately identifying demand in order to achieve accurate service supply and build a perfect community health protection system. Liang-Miin et al.’s paper, “*A Cross-Sectional Study on the Assessment of Service Quality and User Satisfaction of a Community Support Program in a Region of Taiwan*”, focuses on the service quality and user satisfaction of a region-specific Community Support Program (CSP) in Taiwan (Contribution 15). It uses a cross-sectional research design to collect data from 450 CSP users. The study results show that older users, aged 70–79, with primary education, and those with long-term care needs, were the most satisfied with CSP services, highlighting the significant impact of user characteristics and needs on satisfaction. These findings offer clear guidance for policymakers in planning community service development directions, emphasizing the importance of meeting users’ needs, enhancing users’ service awareness, and making the best use of existing service resources. The paper “*Determinants of Self-Rated Health Disparities among Independent Community-Dwelling Older Adults: An Age-Stratified Analysis*”, written by Yuka et al., describes a cross-sectional study conducted on 846 older adults living alone in Yokohama, Japan (Contribution 16). This study similarly showed that for the 75+ age group, self-rated health status was closely linked to economic stability and social participation. These results can offer significant references that Japan and other developed nations can use to formulate targeted healthy aging strategies and enhance the sustainable development of community health management.

When focusing on vulnerable groups, the key factors for success are to enhance their access to resources and remove structural barriers to rehabilitation services. Jung-Lim et al.’s study, “*Status and Barriers of Physical Activity and Exercise in Community*-*Dwelling Stroke Patients in South Korea: A Survey—Based Study*”, used a cross-sectional survey to examine the physical activity and exercise status of 100 community-dwelling stroke patients in South Korea after they had been discharged from hospital (Contribution 17). The study found that there was a “cognitive-activity” gap in the community-based rehabilitation of stroke patients. This gap mainly stemmed from concerns about exercise-related accidents and limited accessibility. Thus, structural improvement measures are required to turn patients’ health awareness into sustainable rehabilitation behaviors. Communities can play a vital role in the community-level management of chronic disease. They can do this by improving community health services, for example by strengthening social infrastructure, ensuring that community staff accompany patients, and developing specialized care programs for patients. These actions can help to create and maintain social connections, care, health, and well-being. Meanwhile, the rehabilitation resources of medical institutions should be extended to the community. By building a service network based on a “hospital–community–family” continuum, the discontinuity in post-discharge support for patients can be reduced. This, in turn, will enhance the quality of life and social participation of stroke patients. Viviane’s study, “*Impact of Prescribed Exercise on the Physical and Cognitive Health of Adults with Down Syndrome: The MinDSets Study*”, empirically explored the effects of a prescribed eight-week exercise and cognitive training intervention on the physical and cognitive health of 83 adults with Down syndrome (DS), in an intervention trial (Contribution 18). The study revealed that walking can create cognitive load via enhanced alertness and decision-making, thereby promoting cognitive health. This finding indicates that community health providers should include walking/jogging as a key part of community health interventions for DS, and include it in the design of comprehensive health support programs.

In addition, another paper highlights the crucial role of cognitive restructuring in eradicating inequalities in community health governance. Egbe et al.’s study, “*Addressing HIV Misconceptions among Heterosexual Black Men and Communities in Ontario*”, sampled 866 heterosexual black men in Ontario, Canada (Contribution 19). This paper offers a foundation for formulating evidence-based strategies. By uncovering the group’s HIV-related misperceptions and identifying protective and risk variables among the social determinants of HIV misperceptions, the study aimed to strengthen HIV prevention efforts and reduce the stigmatization of this group and its communities. The study reveals that HIV cognitive misconceptions are still common among heterosexual Black men, and are closely linked with structural and behavioral factors; to address this, the authors suggest that structural and policy interventions should be carried out. These include improving healthcare access and equity, promoting educational equity, and fostering social inclusion. Also, efforts should be made to enhance collective resilience, promote critical health and racial literacy, and create culturally safe spaces within the community to support intergenerational dialogue.

Collectively, these studies demonstrate that community health governance should thoroughly consider the various stages of the life cycle and the diverse needs of socially disadvantaged groups. By doing so, it can mitigate issues of asymmetric development within community health governance [16]. Additionally, it can facilitate the practical implementation of health equity, transforming it from a conceptual idea into tangible actions, thereby achieving leapfrog development.

## 6. Conclusions

In general, a positive correlation exists between the effectiveness of community health governance and the personal health sustainability of residents. To address the multitude of causal factors involved, co-creating healthcare solutions with the direct involvement of the affected communities is essential [13]. Comprehensively and contextually addressing residents’ needs—regardless of their health status or disease complexity—represents a crucial policy for enhancing population health outcomes [17]. Specifically, efforts should center on empowering residents, strengthening community responsibility, improving cross-sectoral collaborative governance, and balancing social equity based on community disparities. Although significant progress has been made in this field, numerous challenges persist in community health building. For instance, the Tuberculosis Research Institute of Timor-Leste has identified a critical paradox: while medicines can cure diseases, they fail to eliminate social exclusion. This finding underscores the fact that true health sustainability does not primarily depend on clinics or technological applications; rather, it lies in enabling communities to take the lead in identifying their unique needs and formulating contextually appropriate approaches to address them.

The 19 studies presented in this Issue should serve as a compass, rather than a map, guiding us toward a future where power is reconfigured, with each individual community constituting the starting point for developing health initiatives. Future research should further consider community characteristics, institutional environments, ecosystems, and other diverse aspects of development, in order to establish a comprehensive interdisciplinary analytical framework. Policymakers are tasked with strengthening incentives and synergistic mechanisms to drive the systematic construction and promotion of effective community health models [1]. In summary, through multidimensional and cross-disciplinary academic exploration, the research in this field demonstrates the positive impact of community health building on personal sustainable development. We expect that the findings of this research will inspire academics, practitioners, and policymakers to think deeply about community health approaches. Together, we can promote the evolution of community health models, making them more inclusive, resilient, and socially responsible, and ultimately, creating health systems that truly serve the needs of diverse populations.
